# Circular RNA from Tyrosylprotein Sulfotransferase 2 Gene Inhibits Cisplatin Sensitivity in Head and Neck Squamous Cell Carcinoma by Sponging miR-770-5p and Interacting with Nucleolin

**DOI:** 10.3390/cancers15225351

**Published:** 2023-11-09

**Authors:** Tianqing Wang, Chuan Xin, Shiyu Zhang, Xin Tian, Yuting Hu, Ying Wang, Jiongke Wang, Ning Ji, Xin Zeng, Jing Li

**Affiliations:** 1 State Key Laboratory of Oral Diseases & National Center for Stomatology, National Clinical Research Center for Oral Diseases, Research Unit of Oral Carcinogenesis and Management, Chinese Academy of Medical Sciences, West China Hospital of Stomatology, Sichuan University, Chengdu 610041, China; tianqingwang1999@163.com (T.W.); xinchuanr@163.com (C.X.); timecity@163.com (S.Z.); tx18283075716@163.com (X.T.); hyt_1123@163.com (Y.H.); wang_ying2018@163.com (Y.W.); wangjiongke@163.com (J.W.); jining_1023@126.com (N.J.); 2 Key Laboratory of Oral Biomedical Research of Zhejiang Province, School of Stomatology, Zhejiang University School of Medicine, Zhejiang Provincial Clinical Research Center for Oral Diseases, Stomatology Hospital, Cancer Center of Zhejiang University, Hangzhou 310006, China

**Keywords:** head and neck squamous cell cancer, circTPST2, cisplatin sensitivity, miR-770-5p, Nucleolin

## Abstract

**Simple Summary:**

This study explores the role of circTPST2, a circular RNA, in the chemotherapy sensitivity of head and neck squamous cell carcinoma (HNSCC). Elevated levels of circTPST2 were observed in HNSCC tissues, and functional experiments revealed its inhibitory effect on cisplatin sensitivity in HNSCC cells. By interacting with miR-770-5p and the Nucleolin pathway, circTPST2 emerged as a crucial regulator influencing chemotherapy responsiveness. The findings suggest that circTPST2 may serve as a potential marker for chemotherapy regimen selection in HNSCC patients, providing insights to enhance treatment efficacy and patient outcomes.

**Abstract:**

Chemoresistance poses a significant challenge in the treatment of advanced head and neck squamous cell cancer (HNSCC). The role and mechanism of circular RNAs (circRNAs) in HNSCC chemoresistance remain understudied. We conducted circRNA microarray analysis to identify differentially expressed circRNAs in HNSCC. The expression of circRNAs from the tyrosylprotein sulfotransferase 2 (TPST2) gene and miRNAs was evaluated through qPCR, while the circular structure of circTPST2 was verified using Sanger sequencing and RNase R. Through Western blotting, biotin-labeled RNA pulldown, RNA immunoprecipitation, mass spectrometry, and rescue experiments, we discovered miR-770-5p and nucleolin as downstream targets of circTPST2. Functional tests, including CCK8 assays and flow cytometry, assessed the chemoresistance ability of circTPST2, miR-770-5p, and Nucleolin. Additionally, FISH assays determined the subcellular localization of circTPST2, miR-770-5p, and Nucleolin. IHC staining was employed to detect circTPST2 and Nucleolin expression in HNSCC patients. circTPST2 expression was inversely correlated with cisplatin sensitivity in HNSCC cell lines. Remarkably, high circTPST2 expression correlated with lower overall survival rates in chemotherapeutic HNSCC patients. Mechanistically, circTPST2 reduced chemosensitivity through sponge-like adsorption of miR-770-5p and upregulation of the downstream protein Nucleolin in HNSCC cells. The TCGA database revealed improved prognosis for patients with low circTPST2 expression after chemotherapy. Moreover, analysis of HNSCC cohorts demonstrated better prognosis for patients with low Nucleolin protein expression after chemotherapy. We unveil circTPST2 as a circRNA associated with chemoresistance in HNSCC, suggesting its potential as a marker for selecting chemotherapy regimens in HNSCC patients. Further exploration of the downstream targets of circTPST2 advanced our understanding and improved treatment strategies for HNSCC.

## 1. Introduction

Head and neck squamous cell carcinoma (HNSCC), a malignant neoplasm originating from the mucosal epithelium of the oral cavity, pharynx, throat, and sinus, ranks as the sixth most prevalent cancer globally. Despite many efforts, the survival rate of HNSCC patients remains suboptimal, with a 40–50% 5-year survival rate [1]. Chemoradiotherapy is the standard of care for locally advanced HNSCC, serving either as the definitive treatment or as a post-surgical intervention in cases marked by adverse pathological features. The combination of cisplatin and 5-fluorouracil (5-Fu), doxorubicin (Adriamycin, ADR), or cisplatin and docetaxel stands out as the prevailing chemotherapy regimen for HNSCC [2]. Reduced sensitivity to chemotherapeutic drugs emerges as a pivotal factor contributing to treatment failure, culminating in sustained tumor proliferation and metastatic dissemination in HNSCC [3]. Decreased sensitivity to chemotherapeutic drugs is the bottleneck problem faced by current tumor strategies. Current studies have shown that to escape the effects of various chemotherapeutic drugs, tumor cells have evolved a variety of functions, including promoting drug efflux; changing drug targets; enhancing DNA repair capacity, apoptotic escape, and autophagy; and promoting adaptive responses. Consequently, these mechanisms contribute to diminished sensitivity to chemotherapeutic drugs and result in treatment failure. The efficiency of chemotherapeutic drugs is mainly constrained by toxicity to healthy tissues and pharmacokinetic factors (including drug absorption, systemic distribution, elimination, and metabolism). The effects of drugs on tumor cells are called pharmacodynamic properties [4]. It has been reported that tumor pharmacodynamic resistance mechanisms include insufficient drug influx or excessive drug efflux, drug inactivation or lack of activation, changes in drug target expression levels, activation of adaptive survival responses [5], and dysregulation of apoptosis [6]. However, the precise mechanism of chemosensitivity reduction requires further elucidation. Recent in-depth investigations into circular RNAs (circRNAs) have revealed their crucial role not only in tumor proliferation, metastasis, and radiosensitivity but also in the intricate regulation of chemosensitivity across various malignancies.

Circular RNAs, a novel class of endogenous noncoding RNAs that are more stable than linear RNAs, are characterized by their covalently closed-loop structures without a 5′ cap or a 3′ Poly A tail [7]. According to their genomic origin, circRNAs can be broadly categorized into three types: exonic circRNAs, formed by exon skipping or head-to-tail connection; intronic circRNAs, composed of lariat introns; and exon–intron circRNAs, consisting of exons and introns [8]. Exonic circRNAs can reflect the development of diseases and can be used as biomarkers for the diagnosis and prognostic assessment of various diseases [9]. The reported functions of circRNAs include affecting transcription, acting as sponges for adsorbing miRNAs, acting as sponges for proteins, interacting with proteins, and translating into proteins [10]. Numerous studies have demonstrated that circRNAs are involved in the regulation of the chemosensitivity of tumor cells [8]. The circRNA–miRNA pathway and the circRNA–protein pathway are the main mechanisms by which circRNAs exert various functions, including chemosensitivity. For example, related studies on the circRNA–miRNA pathway have found that circRNA EIF6 acts as a sponge for adsorbing miR144-3p to promote cisplatin resistance in human thyroid cancer cells through autophagy [11]; circRNA Cdr1as upregulates cancer cell invasion inhibitors through miR-1270 (suppressor of cancer cell invasion, SCAI), thereby reducing the cisplatin resistance of ovarian cancer [12]. Studies on the circRNA–protein pathway have found that circRNA MTO1 inhibits the resistance of breast cancer cells to monaster by regulating the TRAF4/Eg5 axis [13]; CircPDSS1 confers cisplatin resistance through the miR-515-5p/ITGA11 axis in gastric cancer [14]. Previously, we screened 32 differentially expressed circRNAs by conducting a circRNA microarray in HNSCC, and we validated CircRFWD3’s high expression and promotion of HNSCC metastasis by modulating miR-27a/b/PPARγ signaling [15]. However, to date, there is a paucity of research on the regulatory role of circRNAs in the chemotherapy sensitivity of HNSCC.

In the current study, we discovered that hsa_circ_103188 (termed circTPST2), a circRNA distinctive from the Tyrosylprotein Sulfotransferase 2 (TPST2) gene, is significantly upregulated in HNSCC. miR-770-5p and Nucleolin were found to be downstream target molecules. We also investigated the involvement of these components in chemoresistance to identify prospective markers and possible therapeutic targets to overcome chemoresistance in HNSCC.

## 2. Materials and Methods

### 2.1. Patients and Follow-Up

Data from the cohort comprising 186 HNSCC patients postchemotherapy were obtained from The Cancer Genome Atlas (TCGA) database (Table S1). A total of 69 cases were obtained from chemotherapeutic patients with HNSCC at West China Hospital of Stomatology, Sichuan University, Peking University Stomatology, and Guangzhou Guanghua Dental Hospital. All patients or their family members were followed up for recurrence and prognosis. The starting point of follow-up was the date the patients received surgical treatment, and the recurrence, death, and survival time were recorded. Inclusion criteria: diagnosis of HNSCC confirmed by histopathological examination; chemotherapy after surgery; no other organ tumors; informed consent of patients. The exclusion criteria were as follows: primary tumor with distant metastasis; serious systemic infectious diseases; and pregnancy and lactation. Informed consent was obtained from all patients and approved by the Ethics Committee of Sichuan University. The correlation between clinicopathological features and the expression of Nucleolin was analyzed (Table S2). The study was approved by the ethics committees of the West China Hospital of Stomatology, Sichuan University and adhered to the principles of the Helsinki Declaration. Written informed consent was provided by all participants at baseline and during follow-up.

### 2.2. CircRNA Microarray Analysis

Nine tumor tissues and their corresponding adjacent nontumor tissues were organized into groups of three each for circRNA microarray analysis using Arraystar Human circRNA Array V2 (Arraystar Inc., Rockville, MD, USA). The raw sequencing data of circRNAs have been deposited in GEO (GSE200946). A total of 3157 circRNAs were identified, with 59 showing consistent upregulation and 76 demonstrating downregulation across the three tumor groups compared to nontumor tissues. Candidate circRNAs were further filtered based on the following criteria: 200 bp ≤ base length ≤ 1500; fold change ≥ 2, and *p* < 0.05. After validation in circBase, 32 circRNAs were identified (Table S5).

### 2.3. Cell Cultures

Human oral keratinocyte (HOK) (RRID: CVCL_B404) cells and six human HNSCC cell lines UM1 (RRID: CVCL_VH00), UM2 (RRID: CVCL_VH01), HSC-4 (RRID: CVCL_1289), CAL27 (RRID: CVCL_1107), HN12 (RRID: CVCL_5518), and HN31 (RRID: CVCL_5526) were supplied by the State Key Laboratory of Oral Diseases. HOK cells were cultured in defined keratinocyte SFM medium (10744019, Thermo Fisher Scientific, Waltham, MA, USA). UM1, UM2, HSC-3, CAL27, HN12, and HN31 cells were appropriately cultured in DMEM (SH30243.01, HyClone, Marlborough, MA, USA) supplemented with 10% FBS and 1% penicillin-streptavidin solution. All cells were regularly tested for mycoplasma with a MycAwayTM Plus-Color One-Step Mycoplasma Detection Kit (40612ES25, Yeasen, Shanghai, China) and were authenticated through short tandem repeat profiling and cultured within 2 years. Cells were kept in a humidified incubator at 37 °C with 5% CO_2_.

### 2.4. Transfection Assay

Six-well plates were used to culture HSC-4, HSC-3, UM1, UM2, and HN31 cells, and transfection was carried out when cells were 70–80% confluent. Serum-free medium was used before transfection reagents were added to the corresponding plates. Cells were transfected using Lipofectamine™ RNAiMAX transfection reagent (13778030, Thermo Fisher) following the product’s manual, and cells were seeded after being cultured for 48–72 h. All small interfering RNAs (siRNAs) targeting circTPST2, nucleolin, hnRNPM, miRNA mimics or inhibitors, and negative controls (NCs) were synthesized by RiboBio (Guangzhou, China) (Table S3).

### 2.5. Stable Cell Line Generation

GV493-sh-circTPST2 and CV572-circTPST2 recombinant lentiviruses (sh-circTPST2, sh-NC, OE-circTPST2, and OE-NC), empty GV341 lentivirus, and empty CV572 lentivirus (sh-NC and OE-NC) were purchased from NeuronBiotech (Shanghai Genechem Co., Ltd., Shanghai, China). HSC-4 and HN31 cells were infected with sh-circTPST2 or sh-NC or infected with OE-circTPST2 or OE-NC. A selective culture medium containing 1 µg/mL puromycin was used to select the cells with stable expression of low circTPST2 or vector controls. The expression of circTPST2 was detected by RT–qPCR.

### 2.6. Flow Cytometry

HN31 and HSC-4 cells, which were treated with cisplatin for 48 h after trypsin digestion (note that the trypsin digestion time should be carefully controlled to avoid false positive results), were subjected to flow cytometry after Annexin V-FITC (ab54775, Abcam, Cambridge, UK) and propidium iodide (ab139418, Abcam) apoptosis double staining.

### 2.7. RNA Isolation and qRT–PCR

RNA isolation and qRT–PCR were performed as previously described [15]. Primer sequences are listed in Table S4.

### 2.8. RNase R Digestion Treatment and Sanger Sequencing

RNase R digestion treatment was performed as previously described [15]. Sanger sequencing service was provided by CloudSeq Biotech Inc. (Shanghai, China). Primer sequences for Sanger sequencing were as follows: forward primer: TCACTCAAGCTCATCCTCGA-TCGAGGATGAGCTTGAGTG; reverse primer: CGACAGGTTAGCGGGCA-TGCCCGCTAACCTGTCG.

### 2.9. CCK8 Experiment

After 24 h of cell culture after transfection, the cells were placed in 96-well plates (6 × 10^4^ cells per well) and incubated with cisplatin (Sigma, St. Louis, MO, USA), 5-Fu (Sigma, USA), and ADR (Sigma, USA) at different concentrations (1, 2, 4, 8, 16, 32 mg/mL) for 48 h. Then, 10 µL CCK8 was added to each well, a spectrophotometer was used to detect the absorbance at 450 nm, and the half maximal inhibitory concentration (IC50) of each chemotherapy drug was analyzed in different OSCC cell lines.

### 2.10. Fluorescence In Situ Hybridization (FISH) Assay

RNA fluorescence in situ hybridization (FISH) was performed using the Fluorescent in situ Hybridization Kit (lnc1CM001, RiboBio) following the manufacturer’s guidelines. Labeled probes targeting circTPST2 and U6 were synthesized by RiboBio (Guangzhou, China). Fluorescence was recorded with a confocal laser scanning microscope (FV3000, OLYMPUS).

### 2.11. RNA Pulldown Assay

RNA pulldown assays were performed according to the Pierce™ Magnetic RNA–Protein Pull-Down Kit (20164, Thermo Scientific). Biotinylated circTPST2 probes were synthesized by RiboBio (Guangzhou, China). Pulldown RNA released from the beads after cleansing was evaluated by qRT–PCR. The primer sequences of the biotinylated circTPST2 probes were as follows: circTPST2 probe1: TCACATTTGGACAGGGAGAC; circTPST2 probe2: CCAGGCTCACATTTGGACAG.

### 2.12. Rapid Silver Staining Experiment for Mass Spectrometry

Mass spectrometry was performed using the rapid silver staining kit purchased from Sangon Biotech (Shanghai, China), and WB electrophoresis was carried out on the pulled-down RNA protein. After electrophoresis, the polyacrylamide gel was subjected to a silver staining experiment and then subjected to mass spectrometry for detection.

### 2.13. Western Blot Analysis

For Western blot analysis, cells were washed three times with 1× PBS and then used for extraction of total proteins. Protein extracts were prepared in mammalian lysis buffer. Protein concentrations were measured by the Pierce™ BCA Protein Assay Kit (23250, Thermo Scientific). Protein extracts were separated by 10% SDS–PAGE and transferred onto polyvinylidene fluoride (PVDF) membranes (Millipore, IPVH00010). Then, the PVDF membranes were blotted individually with appropriate primary antibodies against GAPDH (2148 S, Cell Signaling Technology, Danvers, MA, USA, dilution rate 1:3000), cleaved caspase-8 (9661 S, Cell Signaling Technology, dilution rate 1:1000) and caspase-3 (9662 S, Cell Signaling Technology, dilution rate 1:1000), histone H2A. X (9718 S, Cell Signaling Technology, dilution rate 1:1000), tubulin (5335 S, Cell Signaling Technology, dilution rate 1:1000), nucleolin (ab129200, Abcam, dilution rate 1:10,000), hnRNPM (ab226407, Abcam, dilution rate 1:10,000), KPYM (ab150377, Abcam, dilution rate 1:10,000), and appropriate secondary antibodies (mouse, ZB-2305; rabbit, ZB2301, ZSGB-BIO, dilution rate 1:3000). Protein bands were visualized using a chemiluminescence system (Amersham Imager 600). The original Western blotting diagram is presented in the supplementary material (Figures S5–S10).

### 2.14. Immunohistochemistry

Immunohistochemistry (IHC) for Nucleolin (#14574, Cell Signaling Technology, dilution rate 1:400) was performed in specimens after antigen retrieval with citrate buffer (0.01 M, pH 6.0), and then it was visualized by diaminobenzidine (GK600510, DAKO 1:50 dilution). Two experienced pathologists without any knowledge of the clinical and pathological data valued the score of the intensity of staining (0, no staining; 1, weakly stained; 2, moderately stained; 3, strongly stained; 4, extremely stained) and the area of staining (1, 5–25%; 2, 26–50%; 3, 51–75%; 4, >75%). For the statistics of prognostic value in the HNSCC cohort, the total staining score was multiplied by the intensity of staining and the area of staining (1, 2, 3, 4, 6, 8, 9, 12, 16) and was divided into two categories: low expression (the total staining score was 1, 2, 3, 4, 6, 8, and 9) and high expression (the total staining score was 12 and 16).

### 2.15. Statistical Analysis

Statistical analysis. All statistical analyses were carried out using GraphPad Prism version 8.0. Means ± SDs are presented in quantification bar graphs. The data of each group are expressed as the mean ± SD (x ± s). The Shapiro–Wilk test was performed to validate the normal data distribution. The homogeneity of the data variance was verified using the F test, and comparisons between groups for statistical significance were performed with the independent-sample t test, one-way ANOVA, or unpaired two-tailed Student’s *t* test. Correlations were analyzed by the Pearson correlation test. Survival analysis was performed by Kaplan–Meier curves and log-rank tests for significance. *p* values of <0.05 were considered statistically significant. The data are presented as the mean ± SD of three independent experiments, * *p* < 0.05, ** *p* < 0.01, *** *p* < 0.001.

## 3. Results

### 3.1. CircTPST2 Was Identified as a Novel Highly Expressed circRNA in HNSCC

**Figure 1:**
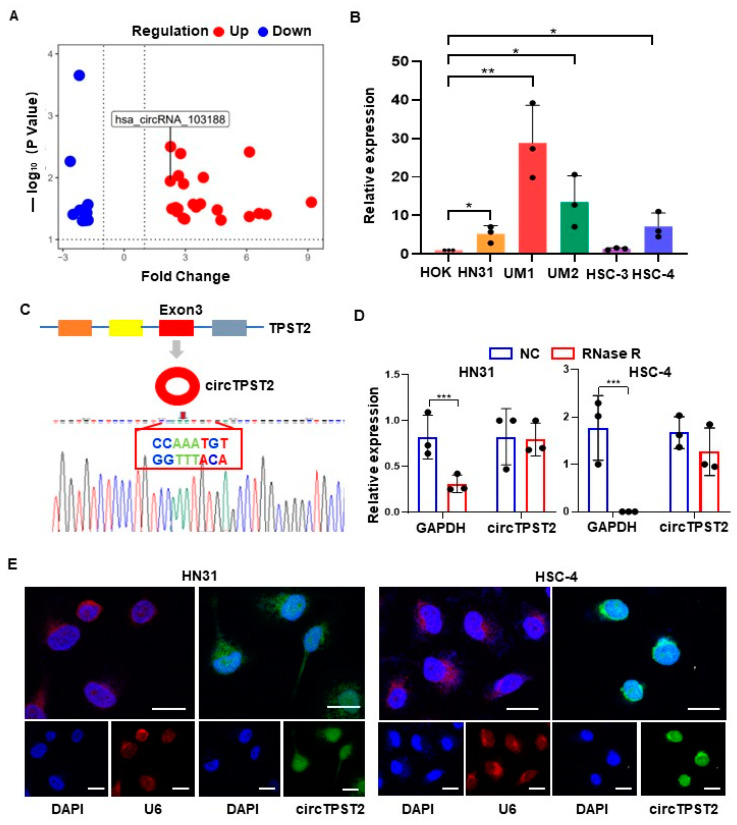
**Expression and characterization of circTPST2 in HNSCC.** (**A**) circRNA microarray analysis was used to detect nine tumor tissues, and the volcano plot shows the differential expression of circRNAs between human HNSCC tissues and adjacent normal tissues. (**B**) qRT–PCR was used to determine the expression level of circTPST2 in normal HOK cells and five HNSCC cell lines (HN31, UM1, UM2, HSC-3, and HSC-4) with the Mann–Whitney test for statistical analysis. (**C**) circTPST2 was primarily transcribed from exon 3 of TPST2, which was measured by qRT–PCR and validated by Sanger sequencing amplified with divergent primers. (**D**) qRT–PCR was used to determine the abundance of circTPST2 and linear TPST2 mRNA in HN31 and HSC-4 cells treated with RNase R. (**E**) RNA FISH showed that circTPST2 was located in the nucleus and cytoplasm compared with the internal reference U6 in HN31 and HSC-4 cells. The nuclei were stained with DAPI (scale bars, 100 μm). Values are expressed as the means ± SDssss; * *p* < 0.05, ** *p* < 0.01, and *** *p* < 0.001.

Our previous report screened 32 differentially expressed circRNAs by conducting a circRNA microarray on nine pairs of cancer and paracancerous tissue analyses in HNSCC [15] (Table S5). Among them, circTPST2 was relatively high in HNSCC tissue compared with adjacent tissues (Figure 1A), and we set circTPST2 as the target circRNA in this study. In vitro experiments showed that circTPST2 is highly expressed in HNSCC cells compared with HOK cells (Figure 1B). CircTPST2 was found to be independently looped by exon 3 of the TPST2 gene by consulting the circBASE and CIRCpedia v2 databases, and Sanger sequencing confirmed that the back-splicing junction site of circTPST2 is CCAAATGT-ACATTTGG (Figure 1C). In addition, the RNase R digestion experiment demonstrated that circTPST2 has a circular structure (Figure 1D). To further determine the localization of circTPST2 in cells, we performed a nucleoplasmic isolation experiment and a fluorescence in situ hybridization (FISH) experiment, and the results showed that circTPST2 was present in both the nucleus and cytoplasm (Figure 1E). The above experimental data proved that circTPST2 is a novel, unique TPST2-derived circRNA that is remarkably upregulated in HNSCC.

### 3.2. The Expression Level of circTPST2 Is Negatively Correlated with the Cisplatin Sensitivity of HNSCC Cells

**Figure 2:**
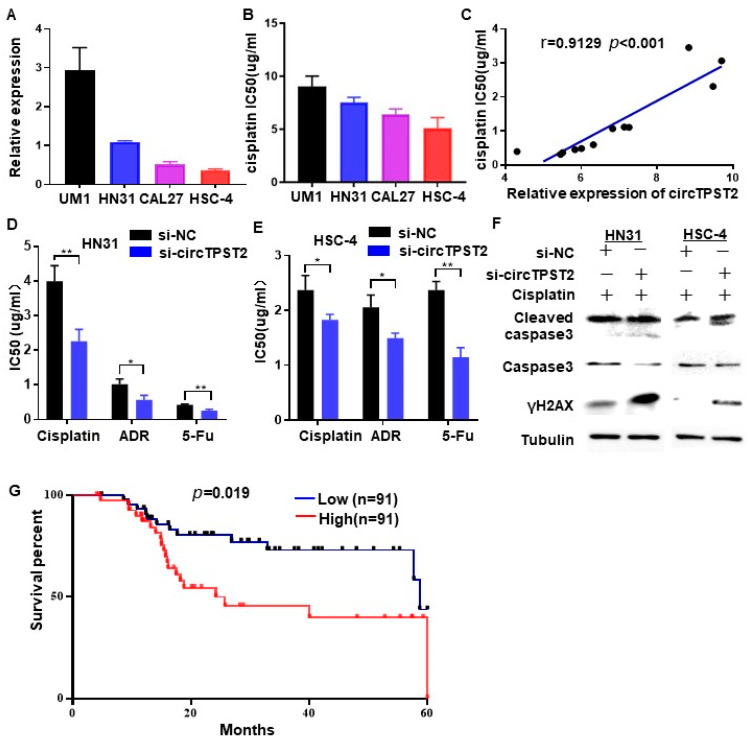
circTPST2 inhibited cisplatin sensitivity in vitro. (**A**) qRT–PCR was used to detect the expression level of circTPST2 in different HNSCC cell lines (UM1, HN31, CAL27, and HSC-4). (**B**) A CCK8 assay was used to test the IC50 of cisplatin in four HNSCC cell lines. (**C**) The expression of circTPST2 was positively correlated with the IC50 of HNSCC cells (r = 0.9129, *p* < 0.001). (**D**,**E**) CCK-8 assay showed that the IC50 of cisplatin, ADR, or 5-Fu in HN31 and HSC-4 cells was significantly reduced after silencing circTPST2. (**F**) Western blot analysis showed that the protein expression of cleaved caspase 3 and γH2AX was evidently increased in two HNSCC cell lines treated with cisplatin for 48 h and transfected with si-circTPST2. The uncropped bolts are shown in Supplementary Materials. (**G**) Kaplan–Meier analysis showed that circTPST2 was negatively correlated with the overall survival rate of HNSCC patients with chemotherapy in the TCGA database (high *n* = 91, low *n* = 91). Values are expressed as the means ± SDssss; * *p* < 0.05, and ** *p* < 0.01.

We predicted the downstream target miRNAs of circTPST2 in the miRBase, miRanda, and TargetScan databases, including miR-593-5p, miR-770-5p, miR-370-3p, miR-637, and miR-383-3p. A literature review found that the downstream miRNAs of cirTPST2 are mostly associated with chemotherapy sensitivity [16,17,18,19]. Thus, we focused on its function in regulating chemoresistance in HNSCC. First, we aimed to uncover the relationship between circTPST2 expression levels and the chemotherapy sensitivity of HNSCC cells. The CCK8 experiment demonstrated that the expression level of circTPST2 was positively related to the IC50 of cisplatin in four HNSCC cell lines, including UM1, HN31, CA27, and HSC-4 (r = 0.9129, *p* < 0.01, Figure 2A–C). We designed three different siRNAs that could specifically target the back-splicing junction site of circTPST2, and si-circTPST-2 was chosen for follow-up experiments because of its efficient inhibition rate (Figure S1A). Si-circTPST-2 was transfected into four HNSCC cell lines, and then the IC50 values of cisplatin, ADR, and 5-Fu in those four HNSCC cell lines were detected. As a result, after knocking down circTPST2, HNSCC cells became more sensitive to chemotherapy drugs (Figure S1B). The sensitivity of HN31 and HSC-4 cells to chemotherapy drugs was significantly increased after silencing the expression of circTPST2, from 39% to 44% and from 23% to 56%, respectively (Figure 2D,E). These two HNSCC cell lines were selected for follow-up experiments. Then, we found that the expression levels of γH2AX and cleaved caspase3 were relatively upregulated in HNSCC cells after circTPST2 was silenced for 48 h by Western blotting (Figure 2F). These results suggest that knocking down circTPST2 increases apoptosis and DNA damage. Moreover, Kaplan–Meier analysis revealed that chemotherapeutic HNSCC patients who expressed circTPST2 at a low level had a longer overall survival (OS) than patients who expressed it at a high level in the TCGA database cohort (*p* = 0.019, Figure 2G). These data distinctly showed that the expression level of circTPST2 is negatively correlated with the chemotherapy drug sensitivity of HNSCC cells.

### 3.3. CircTPST2 Functions as a Sponge for miR-770-5p in HNSCC

**Figure 3:**
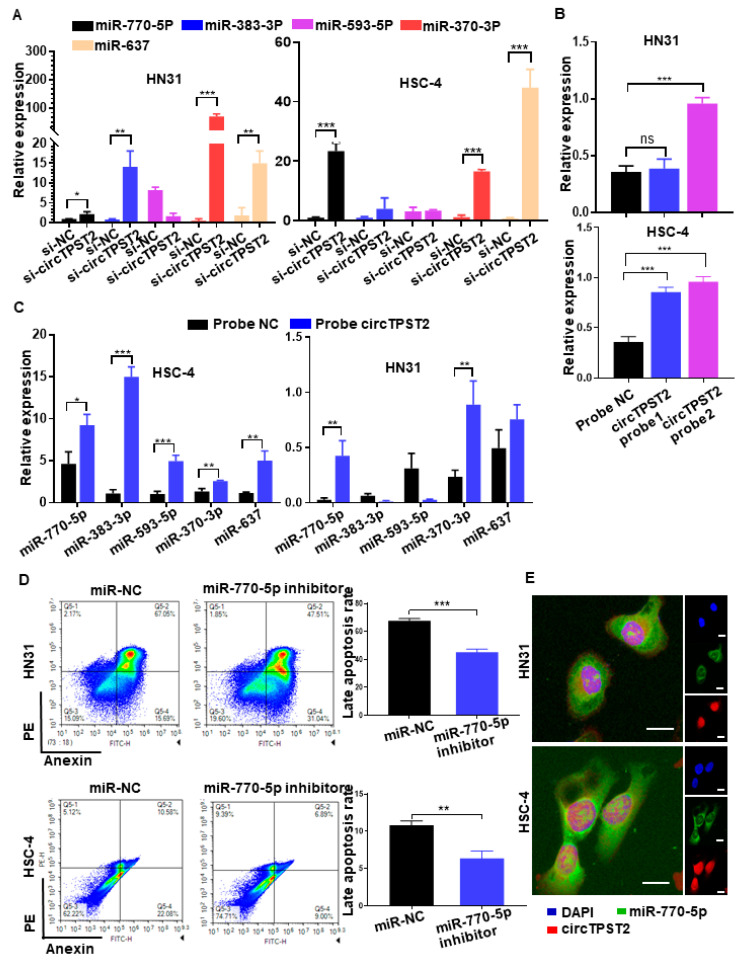
CircTPST2 could act as a sponge of miR-770-5p in HNSCC. (**A**) qRT–PCR assays showed the expression of 5 different types of miRNAs after the transfection of si-NC and si-circTPST2. (**B**) RNA pulldown assays showed that biotinylated circTSPT2 probe 2 could effectively pull down circTSPT2 in both HNSCC cell lines. (**C**) RNA pulldown assays showed that biotinylated circTSPT2 probe 2 could effectively pull down miR-770-5p and miR-370-3p in both HNSCC cell lines. (**D**) A cell flow assay was applied to test the number of late apoptotic cell deaths after the inhibition of miR-770-5p in HNSCC cells. (**E**) RNA FISH showed colocalization of circTPST2 and miR-770-5p in both HNSCC cell lines (scale bar, 100 μm). Values are expressed as the means ± SDssss; * *p* < 0.05, ** *p* < 0.01, and *** *p* < 0.001.

To verify the precise downstream, chemotherapy sensitivity-related miRNAs of circTPST2, we knocked out circTPST2 in HNSCC cells, and then qPCR was carried out to detect the expression levels of five predicted downstream miRNAs (miR-770-5p, miR-383-3p, miR-593-5p, miR-370-3p, and miR-637) (Figure S2A). As a result, elevated levels of miR-770-5p, miR-370-3p, and miR-637 in both cell lines were higher than in the control group (Figure 3A). We constructed a biotinylated probe with the reverse complement of the circTPST2 back-splicing site to verify the mutual interaction between circTPST2 and certain downstream miRNAs. Therefore, it can directly combine with circTPST2. RNA pulldown experiments proved that biotinylated circTPST2 probe 2 has good binding efficiency in both HNSCC cell lines (Figure 3B). Using this probe, we further discovered that circTPST2 can bind to miR-770-5p and miR-370-3p in both HNSCC cell lines (Figure 3C). Cell flow cytometry experiments revealed a significant decrease in advanced apoptotic cells after knocking down miR-770-5p in HNSCC cells (Figure 3D), while no significant differences were observed after knocking down miR-370-3p (Figure S2B). Further immunofluorescence experiments showed that miR-770-5p and circTPST2 could colocalize in both HNSCC cell lines (Figure 3E). Collectively, these experiments demonstrated that miR-770-5p is a precise downstream target of circTPST2, serving as a sponge in HNSCC.

### 3.4. CircTPST2 Physically Interacts with the Nucleolin Protein in HNSCC

**Figure 4:**
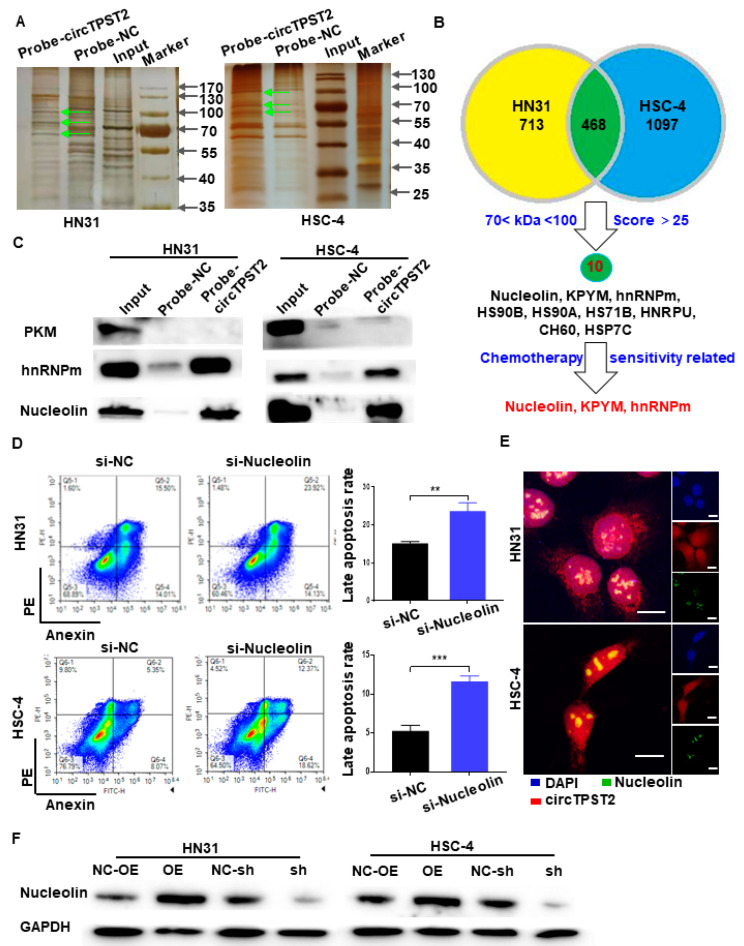
CircTPST2 could interact with the Nucleolin protein in HNSCC. (**A**) Different protein bands detected by silver staining for mass spectrometry of the circTPST2-protein complex pulled down by circTPST2 probe 2 in HN31 and HSC-4 cells. The arrow points to different protein bands. (**B**) Venn diagram showing the overlap of the target circTPST2-binding proteins in both HN31 and HSC-4 cells. (**C**) RNA pulldown and Western blot assays showing that hnRNPm and Nucleolin can interact with circTPST2 but not KPYM in HN31 and HSC-4 cells. (**D**) Flow cytometry assay was used to test the number of late apoptotic cells after the inhibition of Nucleolin expression in HN31 and HSC-4 cells. (**E**) RNA FISH showed colocalization of circTPST2 and miR-770-5p in HN31 and HSC-4 cells (scale bar, 100 μm). (**F**) Western blot showing the expression of Nucleolin in circTPST2 knockdown or circTPST2 overexpression HNSCC cell lines (Values are expressed as the mean ± SD; ** *p* < 0.01 and, *** *p* < 0.001). The uncropped bolts are shown in Supplementary Materials.

To verify the downstream of chemotherapy sensitivity-related proteins of circTPST2, we performed silver-stained polyacrylamide gels on the protein complexes in the RNA pulldown products in two HNSCC cell lines and extracted the proteins in the differential band regions and sent them to mass spectrometry for identification. As a result, three differential bands between the probe-circTPST2 group and the circ-NC group were observed in the range of 70 < protein kDa < 100 (Figure 4A). Mass spectrometry analysis showed that 713 and 1097 candidate proteins bound to circTPST2 were detected in HN31 and HSC-4 cells, respectively. We screened candidate interacting proteins using the following strategy: proteins of the appropriate size, shared by the two HNSCC cell lines, and with the combined Sequest program scored greater than 25. Then, we obtained 10 pending proteins of interest (Figure 4B), namely, heat shock cognate 71 kDa protein (HSP7C), endoplasmic reticulum chaperone BiP (BIP), Nucleolin, heat shock protein HSP 90-beta (HS90B), heat shock 70 kDa protein 1B (HS71B), pyruvate kinase PKM (KPYM), heterogeneous nuclear ribonucleoprotein M (hnRNPm), heat shock protein HSP 90-alpha (HS90A), heterogeneous nuclear ribonucleoprotein U (HNRPU), and 60 kDa heat shock protein (CH60). Among them, three proteins were associated with tumor chemotherapy sensitivity: KPYM, hnRNPm, and Nucleolin. Subsequently, the RNA pulldown assay showed that circTPST2 could pull down hnRNPm and Nucleolin but not KPYM (Figure 4C). This result suggests that circTPST2 can bind to hnRNPm and Nucleolin. Next, to detect the functionality of hnRNPm and Nucleolin in HNSCC, siRNA was used to silence hnRNPm or Nucleolin expression in HSC-4 cells (Figure S3A). Then, we applied flow cytometry assays to reveal that late apoptotic cells increased significantly after inhibiting the expression of Nucleolin (Figure 4D), while late apoptosis in HN31 and HSC-4 cells after inhibition of hnRNPm expression was not noticeably changed (Figure 4D and Figure S3B). FISH experiments showed that circTPST2 and Nucleolin colocalized in HNSCC cells, which further verified that they could interact with each other (Figure 4E). We then constructed stable circTPST2-overexpressing and circTPST2-knockdown HN31 and HSC-4 cell lines (Figure S4). Western blot assays showed that overexpressing circTPST2 increased the expression of Nucleolin, while Nucleolin was less expressed after the knockdown of circTPST2 (Figure 4F). The data above demonstrated that circTPST2 physically interacts with the Nucleolin protein in HNSCC.

### 3.5. CircTPST2 Inhibits Cisplatin Sensitivity by Sponging miR-770-5p and Interacting with Nucleolin

**Figure 5:**
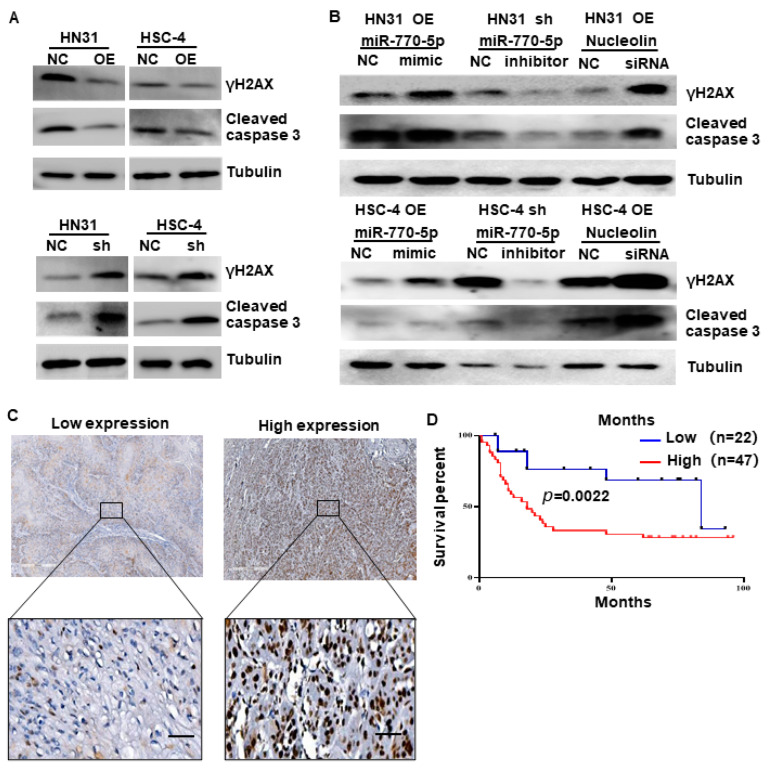
CircTPST2 regulates cisplatin sensitivity via a miR-770-5p and Nucleolin dual mechanism. (**A**) Western blot experiments showed the protein levels of γH2AX and cleaved caspase3 in circTPST2 knockdown and circTPST2 overexpression HNSCC cell lines after cisplatin treatment for 48 h. (**B**) Western blot experiments showed the protein levels of γH2AX and cleaved caspase3 in HNSCC cells with the addition of miR770-5p inhibitors or siRNAs targeting Nucleolin. (**C**) IHC staining of Nucleolin in an HNSCC clinical cohort, scale bar, 10 μm. (**D**) Kaplan–Meier analysis showed that Nucleolin was negatively correlated with the overall survival rate of HNSCC patients with chemotherapy according to our clinical cohort (Values are expressed as the mean ± SD; The uncropped bolts are shown in Supplementary Materials.

**Figure 6:**
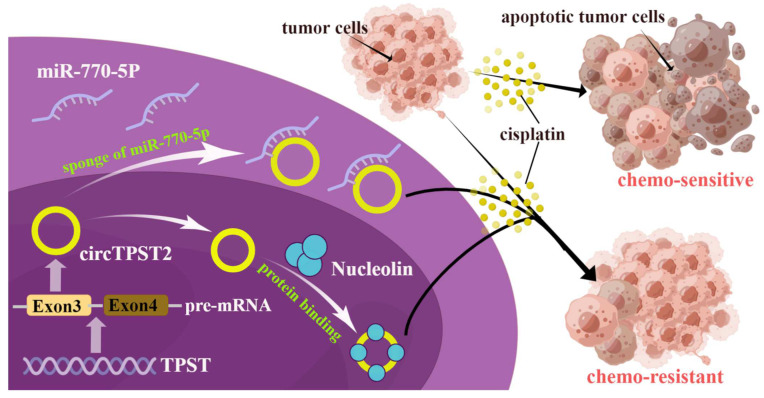
Molecular mechanism by which circTPST2 is involved in chemoresistance in HNSCC. CircTPST2 from exon 3 of TPST pre-mRNA could sponge-like bind to miR-770-5p and interact with Nucleolin to ultimately inhibit cisplatin sensitivity.

To test the function of circTPST2 in chemotherapy, we applied Western blotting and found that the levels of γH2AX and cleaved caspase3 were relatively reduced in HNSCC cells after cisplatin treatment for 48 h, indicating that the expression level of circTPST2 was inversely correlated with apoptosis and DNA damage (Figure 5A). Compared with the control groups, γH2AX and cleaved caspase 3 expression obviously increased in the si-nucleolin group and miR770-5p mimic group (Figure 5B). Then, we obtained a total of 69 cases of chemotherapeutic patients with HNSCC from three different hospitals and assessed the correlation of Nucleolin expression with the prognosis of HNSCC patients by IHC (Figure 5C). Further analysis showed that the protein level of Nucleolin was inversely correlated with prognosis in chemotherapeutic patients with HNSCC (Figure 5D). Taken together, these results suggest that circTPST2 regulates chemotherapy sensitivity by sponging miR-770-5p and interacting with the Nucleolin dual pathway (Figure 6).

## 4. Discussion

CircRNA is a closed-loop structure molecule with covalent bonds that is more stable than linear RNA [20,21]. It was found that circRNAs play important roles in tumor malignancy, including tumor proliferation, metastasis, chemosensitivity, and radiosensitivity [22]. The mechanism of circRNA involvement in tumor chemotherapy sensitivity has been a hot topic in recent years, suggesting the potential of circRNAs as new tools to improve tumor chemosensitivity [23]. However, the role and mechanisms of circRNAs in HNSCC chemosensitivity remain elusive. Here, we identified a novel unique TPST2-derived circTPST2 that is abnormally upregulated in HNSCC tissues and cells. The results of loss- and gain-of-function experiments indicated that circTPST2 inhibits cisplatin sensitivity in HNSCC. Mechanistically, we found that circTPST2 regulates cisplatin sensitivity by sponging miR-770-5p and interacting with the Nucleolin dual pathway in HNSCC.

CircTPST2 is significantly upregulated in HNSCC tissue according to a previous circRNA microarray analysis [15]. In this study, we identified and proved that circTPST2, which is derived from the TPST2 gene, has a ring structure by qPCR, Sanger sequencing, and RNase R. Subsequently, we found that circTPST2 was located in both the nucleus and cytoplasm through immunofluorescence hybridization. Moreover, we found that the expression level of circRNA is negatively correlated with the chemotherapeutic sensitivity of cells, and weakening circTPST2 could enhance the chemosensitivity of HNSCC cells. Additionally, Kaplan–Meier analysis demonstrated that circTPST2 is a risk factor for patient OS, indicating that circTPST2 might function as a chemotherapy regimen selection marker for HNSCC.

Cisplatin-based therapy accounts for up to 50% of tumor chemotherapy, while most patients experience cancer recurrence due to cisplatin resistance [24]. Moreover, circRNAs play an essential role in the mechanism of tumor chemosensitivity [25,26,27,28,29,30,31,32,33,34]. Microarray analysis identified more than 10,000 circRNAs that were dysregulated in cisplatin-resistant cell lines [35]. Growing evidence indicates that circRNAs can act as microRNA sponges and reduce chemoresistance through the circRNA–miRNA–protein signaling pathway [36,37]. For example, circRNA EIF6 acts as a sponge for adsorbing miR-144-3p to promote cisplatin resistance in human thyroid cancer cells through autophagy [38]. Therefore, we speculated that downstream miRNAs or proteins may be related to chemotherapy sensitivity. In this experiment, we chose cisplatin as an appropriate model to investigate the effect of circTPST2 on the sensitivity of HNSCC to chemotherapy. To study the relationship between the expression of circTPST2 and the chemosensitivity of HNSCC, the relationship between the expression of circTPST2 and the IC50 of cisplatin in different HNSCC cells was studied, and it was found that circTPST2 and cisplatin were correlated with the IC50 of HNSCC cells. To investigate whether the content of circTPST2 is widely applicable to other chemotherapeutic drugs (ADR and 5-Fu), we silenced the expression of circTPST2 in HNSCC cells by siRNA and found that the IC50 of chemotherapeutic drugs in five HNSCC cell lines showed a downward trend. This finding indicates that the effect of circTPST2 on the chemosensitivity of HNSCC cells may apply to a variety of chemotherapeutic drugs.

CircRNAs are well known to regulate the tumor process by sponging miRNAs. To reveal the downstream target of circTPST2, we performed an RNA pulldown experiment to find a direct interaction between circTPST2 and miRNAs. We found that inhibition of circTPST2 expression significantly increased intracellular miR-770-5p expression, while inhibition of miR-770-5p significantly attenuated the chemosensitivity of HNSCC cells. Recent studies have demonstrated the crucial role that miR-770-5p plays in cancer and chemoresistance. In ovarian cancer (OVC), miR-770-5p may function as an anti-oncogene and promote cisplatin sensitivity by downregulating ERCC2 in OVC [16]. Another study also indicated that the knockdown of NEAT1 inhibited cisplatin resistance by upregulating miR-770-5p [39]. Additionally, miR-770-5p regulates the resistance of human colorectal adenocarcinoma cells to methotrexate by downregulating HIPK1 [40].

CircRNAs have been shown to control tumor proliferation by interacting with RNA-binding proteins. To better understand the mechanism of circTPST2, we also predicted the possible downstream target proteins of the circTPST2 downstream proteins KPYM, Nucleolin, and hnRNPm by mass spectrometry analysis. The RNA pulldown assay showed that circTPST2 could pull down hnRNPm and Nucleolin but not KPYM. Flow cytometry revealed that inhibiting the expression of Nucleolin markedly increased the number of late apoptotic cells compared to hnRNPm. Nucleolin is important in cancer proliferation, survival, infiltration, and metastasis [41]. Nucleolin is positively regulated by HuR and negatively regulated via competition with miR-494, leading to an obvious reduction in cancer cell survival [42]. Nucleolin has also been reported to mediate cisplatin resistance by stimulating YB1-induced MDR1 transcription [43].

Combining the predictive results, we believe that both miR-770-5p and Nucleolin participate in the regulation of circTPST2-induced chemoresistance. Additionally, the outcomes of a rescue experiment demonstrated that treatment with miR-770-5p mimics or inhibitors could partially reverse the change in the chemoresistance abilities of HNSCC cells caused by circTPST2 overexpression and suppression. Noticeably, the application potential of circTPST2 as a molecular marker of chemotherapy sensitivity in HNSCC patients was discovered using TCGA database analysis. Our study revealed that Nucleolin is negatively correlated with the cisplatin sensitivity of HNSCC cells, and inhibition of Nucleolin can strengthen the chemosensitivity of HNSCC cells.

## 5. Conclusions

In the current study, we identified a distinctive TPST2-derived circRNA that is significantly upregulated in HNSCC tissues and cells and is associated with reduced sensitivity to cisplatin chemotherapy. This suggests that circTPST2 and its downstream target could serve as a biomarker in chemotherapy and a potential treatment target in HNSCC. Collectively, we revealed that circTPST2 inhibits cisplatin sensitivity by sponging miR-770-5p and dually interacting with Nucleolin.

## Data Availability

The data underlying this article will be shared upon reasonable request to the corresponding author.

## Supplementary Materials

The following supporting information can be downloaded at: https://www.mdpi.com/article/10.3390/cancers15225351/s1, Figure S1. The drug sensitivity of HNSCC cells with silenced circTPST2. (A) qRT–PCR assays showed the expression of circTPST2 after simultaneous transfection of si-circTPST2-1, si-circTPST2-2, si-circTPST2-3 and si-NC in HN31 and HSC-4 cells. (B) CCK8 assay was used to test the IC50 of cisplatin, ADR and 5-Fu in four HNSCC cell lines. (Values are expressed as the means ± SDsss; * *p* < 0.05, ** *p* < 0.01 and *** *p* < 0.001); Figure S2. Predictive miRNAs directly bound to circTPST2 and the functional test of miR-370-3p. (A) Schematic illustration showing binding site sequences between circTPST2 and five predicted downstream miRNAs (miR-770-5p, miR-383-3p, miR-593-5p, miR-370-3p, and miR-637). (B) Flow cytometry assay was applied to test the number of late apoptotic cell deaths after the inhibition of miR-370-3p. (Values are expressed as the means ± SDsss; * *p* < 0.05, ** *p* < 0.01 and *** *p* < 0.001); Figure S3. The expression of nucleolin and hnRNPm with silenced circTPST2 and the functional test of hnRNPm. (A) Western blot experiments showed hnRNPm or Nucleolin expression in HSC-4 cells after transfection of siRNA and si-NC. (B) Flow cytometry was used to test the number of late apoptotic cells after the inhibition of hnRNPm expression in HN31 and HSC-4 cells; Figure S4. qPCR was used to test the expression level of circTPST2 in constructed stable circTPST2 knockdown or circTPST2 overexpression cell lines. (Values are expressed as the means ± SDsss; * *p* < 0.05, ** *p* < 0.01 and *** *p* < 0.001); Figure S5. Uncropped whole blots of Figure 2F; Figure S6. Uncropped whole blots of Figure 4C; Figure S7. Uncropped whole blots of Figure 4F; Figure S8. Uncropped whole blots of Figure 5A; Figure S9. Uncropped whole blots of Figure 5B; Figure S10. Uncropped whole blots of Figure S3A; Table S1. The information of 186 patients after chemotherapy for HNSCC from TCGA; Table S2. The correlation between clinicopathological features and expression of nucleolin. Table S3. Sequence of small interfering RNA and miRNA. Table S4. Primer information. Table S5. Normalized intensity of 32 candidate circRNAs.

## Abbreviations

HNSCC, head and neck squamous cell cancer; circRNAs, circular RNAs; miRNAs, MicroRNAs; 5-Fu, 5-fluorouracil; ADR, Adriamycin; TCGA, The Cancer Genome Atlas; HOK, human oral keratinocyte cell; DMEM, Dulbecco’s modified Eagle’s medium; FBS, fetal bovine serum; OE, overexpressing; NC, negative control; qRT–PCR, quantitative real-time reverse transcription PCR; CCK-8, Cell Counting Kit-8; FISH, fluorescence in situ hybridization; PBS, phosphate buffered saline; IHC, immunohistochemistry; SD, standard deviation; HSP7C, heat shock cognate 71 kDa protein; BIP, endoplasmic reticulum chaperone BiP; KPYM, pyruvate kinase PKM; hnRNPm, heterogeneous nuclear ribonucleoprotein M; HS90A, heat shock protein HSP 90-alpha; HS90B, heat shock protein HSP 90-beta; HS71B, heat shock 70 kDa protein 1B; HNRPU, heterogeneous nuclear ribonucleoprotein U; CH60, 60 kDa heat shock protein; PVDF, polyvinylidene fluoride.
