# Supplementary material for: Circular RNA from Tyrosylprotein Sulfotransferase 2 Gene Inhibits Cisplatin Sensitivity in Head and Neck Squamous Cell Carcinoma by Sponging miR-770-5p and Interacting with Nucleolin
Source: Cancers (Basel). 2023 Nov 9;15(22):5351. doi: 10.3390/cancers15225351 (PMC10669990; doi:10.3390/cancers15225351)
Supplement: Supplementary file 1 [file cancers-15-05351-s001.zip › Table S1.pdf]

Table S1. The information of 186 patients after chemotherapy for HNSCC from TCGA

sample	gender	age	OS.time.month	TNM
TCGA.BA.4074.01	MALE	69	15.4	T3N2cM0
TCGA.BA.4075.01	MALE	49	9.433333	T4aN1M0
TCGA.BA.4076.01	MALE	39	13.83333	T3N2cM0
TCGA.BA.4077.01	FEMALE	45	37.8	T4bN3M0
TCGA.BA.5149.01	MALE	47	26.86667	T3N2bM0
TCGA.BA.5153.01	MALE	51	58.73333	T2N1M0
TCGA.BA.5557.01	FEMALE	41	20.76667	T2N2M0
TCGA.BA.6868.01	MALE	53	15.73333	T3N3M0
TCGA.BA.6870.01	FEMALE	60	15.03333	T3N2cM1
TCGA.BA.6872.01	MALE	47	12.8	T3N2cM0
TCGA.BA.6873.01	MALE	28	4.066667	T2N2bM0
TCGA.BA.A4IF.01	MALE	59	29.83333	T4N0M0
TCGA.BA.A4IG.01	MALE	77	28.5	NA
TCGA.BA.A4IH.01	MALE	57	20.73333	T2N2bM0
TCGA.BA.A6D8.01	MALE	59	28.33333	T4aN2cM0
TCGA.BA.A6DA.01	FEMALE	41	11.7	T4aN2cM0
TCGA.BA.A6DD.01	MALE	44	NA	T4aN2cM0
TCGA.BA.A6DI.01	MALE	62	NA	T3N0M0
TCGA.BA.A6DJ.01	MALE	62	13.56667	T4N2bM0
TCGA.BA.A6DL.01	MALE	59	20.76667	T3N0M0
TCGA.BA.A8YP.01	MALE	50	16.63333	T4N3M0
TCGA.BB.4223.01	MALE	48	107.3667	T3N2bM0
TCGA.BB.4224.01	MALE	52	NA	T3N0M0
TCGA.BB.4225.01	MALE	73	4.866667	T3N2cM0
TCGA.BB.4227.01	MALE	66	4.466667	T4aN2bM0
TCGA.BB.7861.01	MALE	56	22.73333	TXN1M0
TCGA.BB.7864.01	MALE	61	50.9	T3N2cM0
TCGA.BB.7866.01	MALE	40	45.6	T3N1M0
TCGA.BB.7872.01	MALE	63	38.93333	T2N2cM0
TCGA.BB.A5HU.01	MALE	47	26.06667	NA
TCGA.BB.A5HY.01	MALE	64	10.7	T3N2bM0
TCGA.BB.A6UM.01	MALE	52	13.1	T2N2bMX
TCGA.CN.4726.01	MALE	68	4.733333	T3N2M0
TCGA.CN.4727.01	MALE	56	52	T4aN0M0
TCGA.CN.4728.01	MALE	56	57.46667	T2N2M0
TCGA.CN.4731.01	FEMALE	63	33.26667	T4aN2M0
TCGA.CN.4735.01	MALE	52	57.9	T3N2bM0
TCGA.CN.4736.01	FEMALE	70	13.16667	T2N0M0
TCGA.CN.4737.01	MALE	19	20.83333	T2N0M0
TCGA.CN.4739.01	MALE	71	46.46667	T4aN0M0

TCGA.CN.4740.01	FEMALE	79	27.96667	T4aN0M0
TCGA.CN.4741.01	MALE	75	74.63333	T4aN0M0
TCGA.CN.4742.01	FEMALE	48	13.23333	T4aN1M0
TCGA.CN.5358.01	MALE	60	8.7	T2N1M0
TCGA.CN.5359.01	MALE	59	12.56667	T4aN1M0
TCGA.CN.5360.01	MALE	68	72.3	T4aN0M0
TCGA.CN.5364.01	MALE	55	16.43333	T4aN2cM0
TCGA.CN.5366.01	MALE	51	12	T4aN2cM0
TCGA.CN.5367.01	FEMALE	60	11.73333	T4aN0M0
TCGA.CN.5370.01	MALE	78	8.633333	T2N0M0
TCGA.CN.5374.01	FEMALE	56	57.73333	T2N2M0
TCGA.CN.6010.01	MALE	53	50.76667	T4aN0M0
TCGA.CN.6011.01	MALE	57	31.1	T4aN0M0
TCGA.CN.6012.01	MALE	66	48.66667	T3N1M0
TCGA.CN.6013.01	MALE	56	24.23333	T4aN2bM0
TCGA.CN.6016.01	MALE	64	48.1	T4aN1M0
TCGA.CN.6017.01	MALE	55	28.43333	T2N1M0
TCGA.CN.6019.01	MALE	61	34.6	T4aN0M0
TCGA.CN.6023.01	MALE	73	52.8	T4aN0M0
TCGA.CN.6989.01	MALE	64	32.66667	T3N2bM0
TCGA.CN.6992.01	MALE	61	35.53333	T4aN1M0
TCGA.CN.6996.01	FEMALE	58	17.66667	T3N2bM0
TCGA.CN.6997.01	MALE	66	32.93333	T4aN2M0
TCGA.CN.6998.01	MALE	53	11.9	T4aN1M0
TCGA.CN.A498.01	FEMALE	61	25.76667	T2N0M0
TCGA.CN.A49A.01	MALE	60	17.53333	T4aN0M0
TCGA.CN.A49C.01	MALE	67	21.5	T4aN2bM0
TCGA.CN.A63T.01	MALE	60	7.5	T3N2bM0
TCGA.CN.A63W.01	FEMALE	48	12.56667	T4aN2cM0
TCGA.CN.A641.01	MALE	47	12.23333	T4N2cM0
TCGA.CN.A6UY.01	MALE	57	23.76667	T3N2aM0
TCGA.CN.A6V1.01	MALE	59	20.1	NA
TCGA.CN.A6V3.01	MALE	61	24.73333	NA
TCGA.CN.A6V6.01	MALE	59	21.16667	T2N2bM0
TCGA.CN.A6V7.01	MALE	40	19.8	T2N2bM0
TCGA.CQ.5327.01	FEMALE	61	55.33333	T3N2cM0
TCGA.CQ.5330.01	FEMALE	69	63.23333	T3N1M0
TCGA.CQ.6218.01	FEMALE	52	41.76667	T3N0M0
TCGA.CQ.6222.01	MALE	63	67.2	T3N2bM0
TCGA.CQ.6224.01	MALE	52	57.36667	T2N0M0
TCGA.CQ.7065.01	MALE	40	54.26667	T2N0M0
TCGA.CQ.A4CH.01	MALE	58	12.63333	T4aN2cM0
TCGA.CR.5243.01	MALE	51	85.4	T3N2bM0

TCGA.CR.5247.01	MALE	48	11.93333	T3N1M0
TCGA.CR.5248.01	MALE	53	55.43333	T4aN2cM0
TCGA.CR.5249.01	FEMALE	35	38.4	T2N0M0
TCGA.CR.5250.01	MALE	71	26.63333	T2N0M0
TCGA.CR.6470.01	MALE	38	50.7	T1N2cM0
TCGA.CR.6471.01	MALE	58	40.06667	T4aN0M0
TCGA.CR.6472.01	MALE	59	35	T2N3M0
TCGA.CR.6473.01	MALE	68	37.5	T3N2bM0
TCGA.CR.6474.01	MALE	51	18.8	T3N2aM0
TCGA.CR.6477.01	FEMALE	56	17.13333	T3N2bM0
TCGA.CR.6478.01	FEMALE	66	6.1	T3N2cM0
TCGA.CR.6480.01	MALE	53	12.06667	T2N2aM0
TCGA.CR.6481.01	MALE	47	10.36667	T2N2aM0
TCGA.CR.6482.01	MALE	62	11.5	T2N2bM0
TCGA.CR.6484.01	FEMALE	67	11.8	T4aN0M0
TCGA.CR.6491.01	MALE	60	23.1	T4aNXM0
TCGA.CR.6493.01	MALE	69	9.4	T3N2bM0
TCGA.CR.7365.01	MALE	60	39.7	T4aN2bM0
TCGA.CR.7367.01	MALE	52	48	T4aN3M0
TCGA.CR.7368.01	MALE	54	41.5	T4aN0M0
TCGA.CR.7373.01	MALE	66	29.63333	T4aN2bM0
TCGA.CR.7380.01	MALE	58	20.2	T3N0M0
TCGA.CR.7382.01	MALE	49	26.53333	T2N2cM0
TCGA.CR.7383.01	FEMALE	79	17.36667	T1N0M0
TCGA.CR.7385.01	MALE	42	33.23333	T2N2bM0
TCGA.CR.7386.01	MALE	69	47.66667	T4aN1M0
TCGA.CR.7388.01	FEMALE	70	27.43333	T2N2M0
TCGA.CR.7389.01	MALE	55	13.06667	T2N1M0
TCGA.CR.7393.01	MALE	26	33.1	T1N0M0
TCGA.CR.7397.01	MALE	44	25.13333	T3N2bM0
TCGA.CR.7402.01	MALE	68	30.36667	T3N0M0
TCGA.CR.7404.01	MALE	53	49.06667	T3N2bM0
TCGA.CV.5430.01	MALE	61	141.3667	T4aN2bM0
TCGA.CV.5434.01	MALE	60	110.4667	T4aN1M0
TCGA.CV.5435.01	MALE	57	77.3	T4aN0M0
TCGA.CV.5439.01	MALE	62	18.2	T2N0M0
TCGA.CV.5440.01	MALE	52	109	T4aN2bM0
TCGA.CV.7416.01	FEMALE	29	NA	T4aN0M0
TCGA.D6.6517.01	MALE	59	9.733333	T3N0M0
TCGA.D6.A6EK.01	MALE	67	29.16667	T4N1M0
TCGA.DQ.5624.01	FEMALE	43	59.26667	T4aN1M0
TCGA.DQ.5629.01	MALE	64	31.36667	T4aN2cM0
TCGA.DQ.5631.01	MALE	52	18.26667	T3N2bM0

TCGA.DQ.7588.01	MALE	66	14.23333	T2N1M0
TCGA.DQ.7589.01	MALE	70	46.96667	T2N2cM1
TCGA.DQ.7590.01	MALE	51	47.1	T1N2bM0
TCGA.DQ.7591.01	MALE	62	20.73333	T4aN2bM0
TCGA.DQ.7592.01	MALE	57	38.1	T4aN2bM0
TCGA.DQ.7593.01	MALE	58	40.8	T4aN1M0
TCGA.DQ.7594.01	MALE	47	40.6	T4aN3M0
TCGA.DQ.7595.01	MALE	53	39.66667	T2N0M0
TCGA.DQ.7596.01	MALE	48	42.16667	T2N2aM0
TCGA.F7.A50J.01	FEMALE	67	31.56667	T3N0M0
TCGA.H7.8501.01	MALE	54	15.36667	T4aN2aM0
TCGA.H7.8502.01	MALE	50	15.26667	T4aN2bM0
TCGA.H7.A6C4.01	FEMALE	35	NA	NA
TCGA.HD.7229.01	MALE	60	34.23333	T4aNXM0
TCGA.HD.7753.01	MALE	62	28.86667	T2N0M0
TCGA.HD.8224.01	MALE	63	14.86667	T3N0M0
TCGA.HD.8314.01	MALE	58	22.33333	T1N1M0
TCGA.HL.7533.01	MALE	65	35.23333	T2N2bM0
TCGA.IQ.7630.01	MALE	49	16.16667	T3N0M0
TCGA.IQ.A61E.01	FEMALE	55	38.23333	T3N0MX
TCGA.IQ.A61G.01	MALE	57	12	T4aN2cMX
TCGA.IQ.A61J.01	MALE	54	34.03333	T2N2bM0
TCGA.IQ.A61K.01	FEMALE	70	NA	T3N2bM0
TCGA.IQ.A61L.01	FEMALE	72	13.86667	T2N0M0
TCGA.IQ.A61O.01	MALE	43	14.03333	NA
TCGA.KU.A6H7.01	FEMALE	55	19.53333	T2N2aM0
TCGA.KU.A6H8.01	MALE	41	10.9	T1N0M0
TCGA.MT.A67G.01	FEMALE	53	6.333333	T1N2bMX
TCGA.MZ.A5BI.01	MALE	53	NA	T2N2bM0
TCGA.MZ.A6I9.01	MALE	68	16.3	T4aN2bM0
TCGA.MZ.A7D7.01	MALE	51	18.23333	T3N2bM0
TCGA.P3.A5Q6.01	MALE	49	16	T3N0M0
TCGA.P3.A5QF.01	MALE	49	NA	T4N2bM0
TCGA.P3.A6SW.01	MALE	50	NA	T4N2bM0
TCGA.P3.A6SX.01	MALE	67	NA	T4N0M0
TCGA.P3.A6T2.01	MALE	45	76.6	T2N0M0
TCGA.P3.A6T3.01	MALE	49	NA	T4N1M0
TCGA.P3.A6T6.01	MALE	53	13.16667	T4N0M0
TCGA.P3.A6T7.01	MALE	55	NA	T3N1M0
TCGA.P3.A6T8.01	MALE	54	NA	T4aN0M0
TCGA.QK.A6IF.01	MALE	61	23.46667	T1N2bM0
TCGA.QK.A6IG.01	MALE	69	7.4	T2N0M0
TCGA.QK.A6IH.01	FEMALE	65	21.76667	T4aN1M0

TCGA.QK.A6II.01	MALE	52	9.466667	T3N0M0
TCGA.QK.A8Z7.01	MALE	59	13.06667	T4aN2cM0
TCGA.QK.A8Z8.01	FEMALE	60	NA	T3N1M1
TCGA.QK.A8Z9.01	MALE	56	14.96667	T4aN2bM0
TCGA.QK.A8ZA.01	MALE	60	12.36667	T2N2bMX
TCGA.QK.AA3K.01	MALE	60	8.433333	T3N2bMX
TCGA.RS.A6TO.01	FEMALE	82	12.9	T4N2cM0
TCGA.T2.A6WX.01	FEMALE	73	NA	T3N0M0
TCGA.T2.A6WZ.01	MALE	53	16.13333	T3N2bM0
TCGA.T2.A6X0.01	MALE	49	NA	T1N2aM0
TCGA.TN.A7HJ.01	MALE	51	13.43333	T3NXM0
TCGA.TN.A7HL.01	MALE	59	20.63333	T4aNXM0
TCGA.UF.A7JC.01	MALE	42	18.2	T3N1M0
TCGA.UF.A7JF.01	MALE	80	56.2	T4aN2bM0
TCGA.UF.A7JJ.01	MALE	68	18.3	T4aN0M0
TCGA.UF.A7JK.01	MALE	59	NA	T4aN0M0
TCGA.UF.A7JT.01	FEMALE	72	NA	T4aN0M0
