# Supplementary material for: Circular RNA from Tyrosylprotein Sulfotransferase 2 Gene Inhibits Cisplatin Sensitivity in Head and Neck Squamous Cell Carcinoma by Sponging miR-770-5p and Interacting with Nucleolin
Source: Cancers (Basel). 2023 Nov 9;15(22):5351. doi: 10.3390/cancers15225351 (PMC10669990; doi:10.3390/cancers15225351)
Supplement: Supplementary file 1 [file cancers-15-05351-s001.zip › Table S2.pdf]

**Table S2. The correlation between clinicopathological features and expression of nucleolin**

| Characteristics | nucleolin high expression (n=22) | nucleolin low expression (n=47) | P value |
|---|---|---|---|
| Gender | | | 0.8011 |
| Male | 17 | 35 | |
| Female | 5 | 12 | |
| Ages | | | 0.1422 |
| ≤60 | 8 | 26 | |
| > 60 | 14 | 21 | |
| Smoking | | | 0.9163 |
| Yes | 12 | 25 | |
| No | 10 | 22 | |
| Tumor differentiation | | | 0.8504 |
| 1 | 14 | 31 | |
| 2-3 | 8 | 16 | |
| T stage | | | 0.2397 |
| T1+T2 | 15 | 25 | |
| T3+T4 | 7 | 22 | |
| N stage | | | 0.2538 |
| N0 | 8 | 24 | |
| N1+N2 | 14 | 23 | |

Data were compared using chi-square test or Fisher's test
