# Supplementary material for: Circular RNA from Tyrosylprotein Sulfotransferase 2 Gene Inhibits Cisplatin Sensitivity in Head and Neck Squamous Cell Carcinoma by Sponging miR-770-5p and Interacting with Nucleolin
Source: Cancers (Basel). 2023 Nov 9;15(22):5351. doi: 10.3390/cancers15225351 (PMC10669990; doi:10.3390/cancers15225351)
Supplement: Supplementary file 1 [file cancers-15-05351-s001.zip › Table S3.pdf]

Table S3. Sequence of small interfering RNA and miRNA

Gene	Sequence
si-circTPST2-1:	TGTCTCCCTGTCCAAATGT
si-circTPST2-2:	TCTCCCTGTCCAAATGTGA
si-circTPST2-3:	CCTGTCCAAATGTGAGCCT
siRNA-hnRNPM-1:	GGTGTGGCGTGGTTAAGTT
siRNA-hnRNPM-2:	GAGAGATTGACGTTCGAAT
siRNA-hnRNPM-3:	GAAGGCCTGCCAGATATTT
siRNA-nucleolin-1:	GAACCGACTACGGCTTTCA
siRNA-nucleolin-2:	GTCGTCATACCTCAGAAGA
siRNA-nucleolin-3:	CGTTCGGGCAAGGATAGTT
miR-770-5p	UCCAGUACCACGUGUCAGGGCCA
