# Supplementary material for: Circular RNA from Tyrosylprotein Sulfotransferase 2 Gene Inhibits Cisplatin Sensitivity in Head and Neck Squamous Cell Carcinoma by Sponging miR-770-5p and Interacting with Nucleolin
Source: Cancers (Basel). 2023 Nov 9;15(22):5351. doi: 10.3390/cancers15225351 (PMC10669990; doi:10.3390/cancers15225351)
Supplement: Supplementary file 1 [file cancers-15-05351-s001.zip › Table S4.pdf]

Table S4. Primer information

Gene	Forward primer (5’→3’)	Reverse primer (5’→3’)
divergent primer		
GAPDH	GAAGGTGAAGGTCGAGTC	GAAGATGGTGATGGGATTTC
circTPST2	TGCATGGAGGTAGGCAAGG	GGGCTTCAGCGACAGGTTAG
convergent primer		
GAPDH	CCATCACCATCTTCCAGGAG-	ATGATGACCCTTTTGGCTCC
circTPST2	CATGGAGGTAGGCAAGGAGAA	GGCTCACATTTGGACAGGGA
U6	CAGCACATATACTAAAATTGGAACG	ACGAATTTGCGTGTCATCC
hsa-miR-593-5p	AGGCACCAGCCAGGCAT	GTGCAGGGTCCGAGGT
hsa-miR-770-5p	TTGCTCCAGTACCACGTGTCA	GTGCAGGGTCCGAGGT
hsa-miR370-3p	AACTTGCCTGCTGGGGTG	TATGGTTGTTCACGACTCCTTCAC
hsa-miR-637	CCACATTACTGGGGGCTTTCG	TATGGTTGTTCTCGACTCCTTCAC
hsa-miR-383-3p	ACACACTGTCACAGCACTGCC	TATCCTTGTTCACGACTCCTTCAC
