# Supplementary material for: Circular RNA from Tyrosylprotein Sulfotransferase 2 Gene Inhibits Cisplatin Sensitivity in Head and Neck Squamous Cell Carcinoma by Sponging miR-770-5p and Interacting with Nucleolin
Source: Cancers (Basel). 2023 Nov 9;15(22):5351. doi: 10.3390/cancers15225351 (PMC10669990; doi:10.3390/cancers15225351)
Supplement: Supplementary file 1 [file cancers-15-05351-s001.zip › Table S5.pdf]

Table S5. Normalized intensity of 32 candidate circRNAs

	Normalized Intensity					
circRNA	TA	CA	TB	CB	TC	CC
hsa_circRNA_400059	9.263587155	6.417325586	8.804030248	4.593081485	8.683469943	6.149112554
hsa_circRNA_400031	7.924627124	6.266201432	10.10182528	6.802651347	10.31057731	6.872033881
hsa_circRNA_104762	9.818798557	6.802651347	8.56085037	6.894439191	9.39689467	5.906139663
hsa_circRNA_104616	9.651294712	6.826151567	8.02865448	5.731602637	8.24500806	5.512082875
hsa_circRNA_102513	11.23788838	12.23788838	12.63044964	9.570112697	12.68496088	9.404010649
hsa_circRNA_101877	10.49831947	9.11972477	9.188923691	6.984537451	9.927361354	6.769975918
hsa_circRNA_001067	13.33375664	11.90098078	13.39327453	11.08998145	12.86656457	10.02958923
hsa_circRNA_400103	9.339223759	7.208336252	7.99181722	5.817250881	8.628635497	7.063957068
hsa_circRNA_104598	10.2636238	8.838842894	8.745289255	6.244166122	9.586781212	7.80285308
hsa_circRNA_104166	9.691252846	7.335855793	8.724691574	7.481547414	9.055649691	7.197728904
hsa_circRNA_101744	10.26577377	8.903787805	10.23018313	8.649143983	10.4566646	8.12223498
hsa_circRNA_100542	9.212217776	7.554349235	8.9952078	7.623043382	8.943400116	7.564546689
hsa_circRNA_103595	8.064125382	6.434107391	7.442684598	6.28328183	8.050585738	6.596966605
hsa_circRNA_100641	9.85974511	8.026811737	9.524807006	8.577940269	9.907344539	8.491059841
hsa_circRNA_104700	12.15760024	10.86738334	12.64643918	10.77900969	11.00653795	10.045262
hsa_circRNA_103401	9.560910009	7.798539483	8.983379699	8.0451341	9.028280287	7.71746242
hsa_circRNA_103563	13.16194049	11.97905671	13.56158256	12.69605515	13.88505782	12.24648393
hsa_circRNA_101711	10.84951101	9.788123589	10.85380501	9.637172072	10.65386207	9.365889584
hsa_circRNA_101379	9.727300106	11.08434476	7.490266149	9.086937942	8.139391612	9.383996785
hsa_circRNA_103915	6.076148121	7.898664531	6.076148121	7.898664531	5.784908163	6.68372608
hsa_circRNA_104067	6.195782842	7.348451783	6.240990565	7.341055702	5.961209143	7.10993974
hsa_circRNA_103704	6.857592631	7.946236336	5.767357588	7.264931495	5.413981389	6.18590838
hsa_circRNA_100783	7.876325256	9.378588447	7.858305012	8.649143983	7.520045897	8.306678951
hsa_circRNA_102285	8.138111918	9.470425918	6.802651347	7.570883689	7.34918931	7.995590669
hsa_circRNA_102347	6.631329058	7.688614774	6.631329058	7.688614774	6.763351076	7.338086621
hsa_circRNA_103917	6.40034627	7.490266149	6.894439191	7.51676723	6.429930205	7.191162328
hsa_circRNA_104236	8.628939214	9.759558857	7.30349563	8.146197542	7.631556226	8.113579542
hsa_circRNA_102634	7.132392906	7.667481386	5.999296012	6.961570597	6.287905238	7.393480079
hsa_circRNA_103188	14.03462436	12.77902891	14.24095046	13.30686421	14.53397667	13.17961919
hsa_circRNA_104503	12.0852961	10.76235611	11.77051336	10.35412312	11.50472074	9.617410119
hsa_circRNA_101740	8.345636375	6.651799561	8.20604786	7.320142657	8.265077924	6.240990565
hsa_circRNA_101852	11.14983312	8.990015379	9.423400521	8.479654084	10.84298049	9.232745753
