# Supplementary figures and images for: Circular RNA from Tyrosylprotein Sulfotransferase 2 Gene Inhibits Cisplatin Sensitivity in Head and Neck Squamous Cell Carcinoma by Sponging miR-770-5p and Interacting with Nucleolin
Source: Cancers (Basel). 2023 Nov 9;15(22):5351. doi: 10.3390/cancers15225351 (PMC10669990; doi:10.3390/cancers15225351)

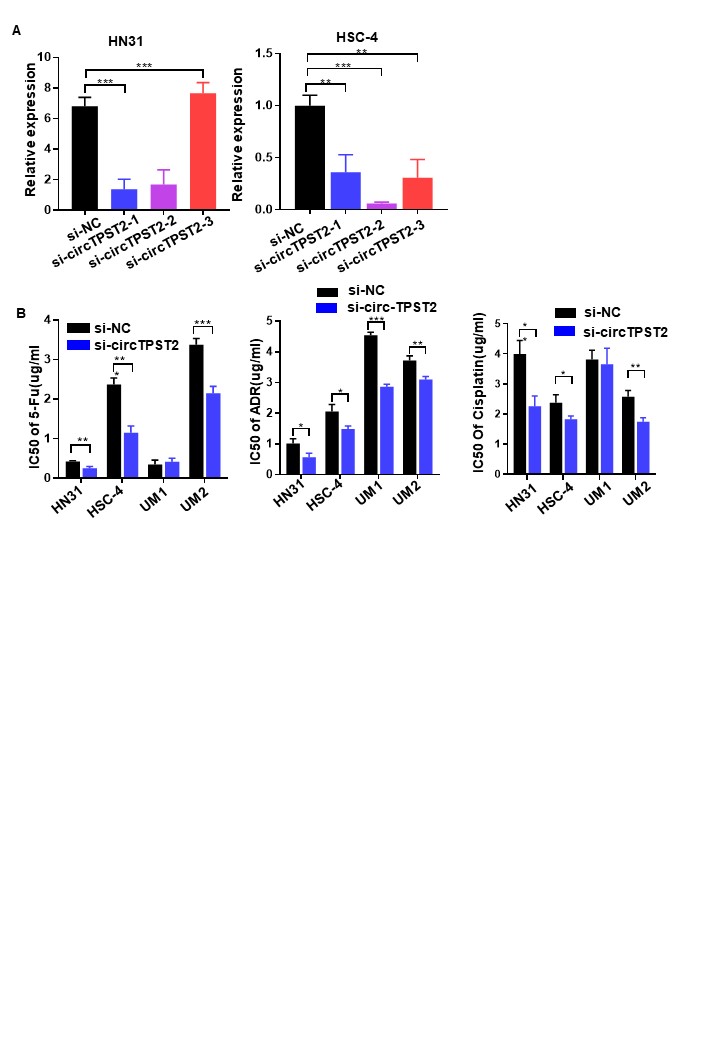

Supplement: Supplementary file 1 [file cancers-15-05351-s001.zip › Figure S1.JPG]

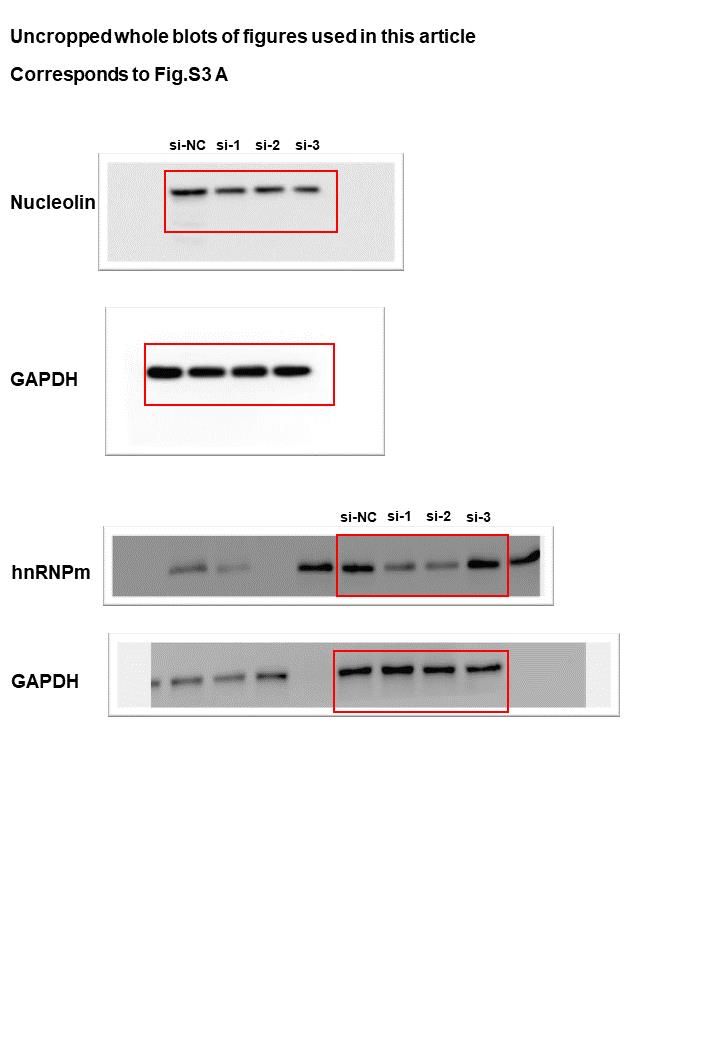

Supplement: Supplementary file 1 [file cancers-15-05351-s001.zip › Figure S10.jpg]

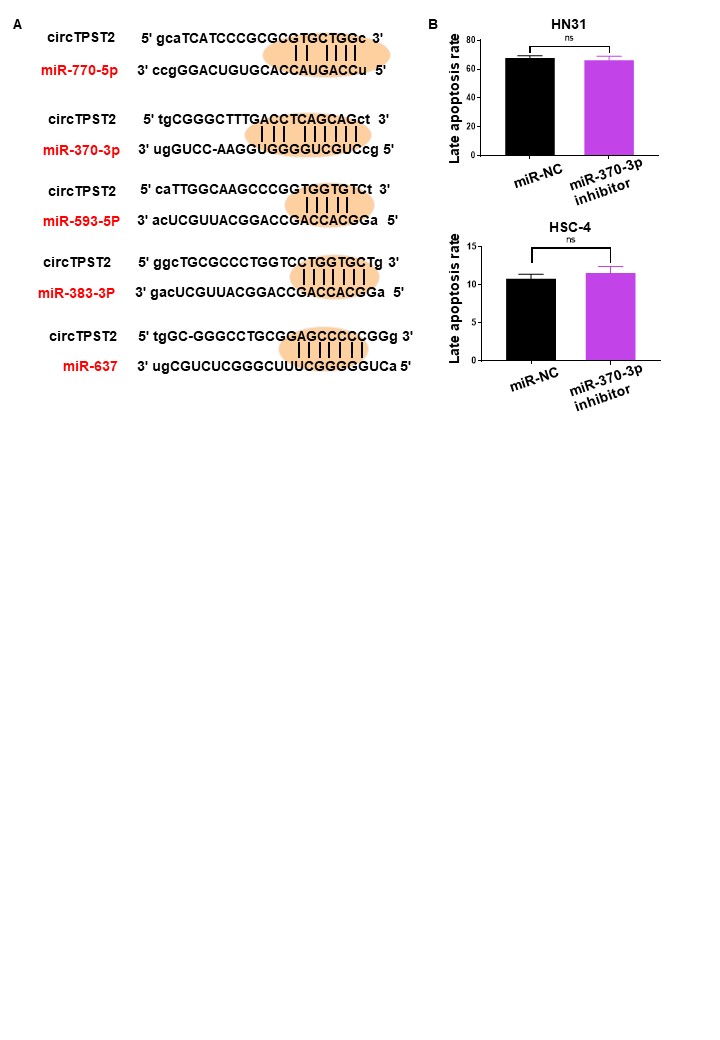

Supplement: Supplementary file 1 [file cancers-15-05351-s001.zip › Figure S2.JPG]

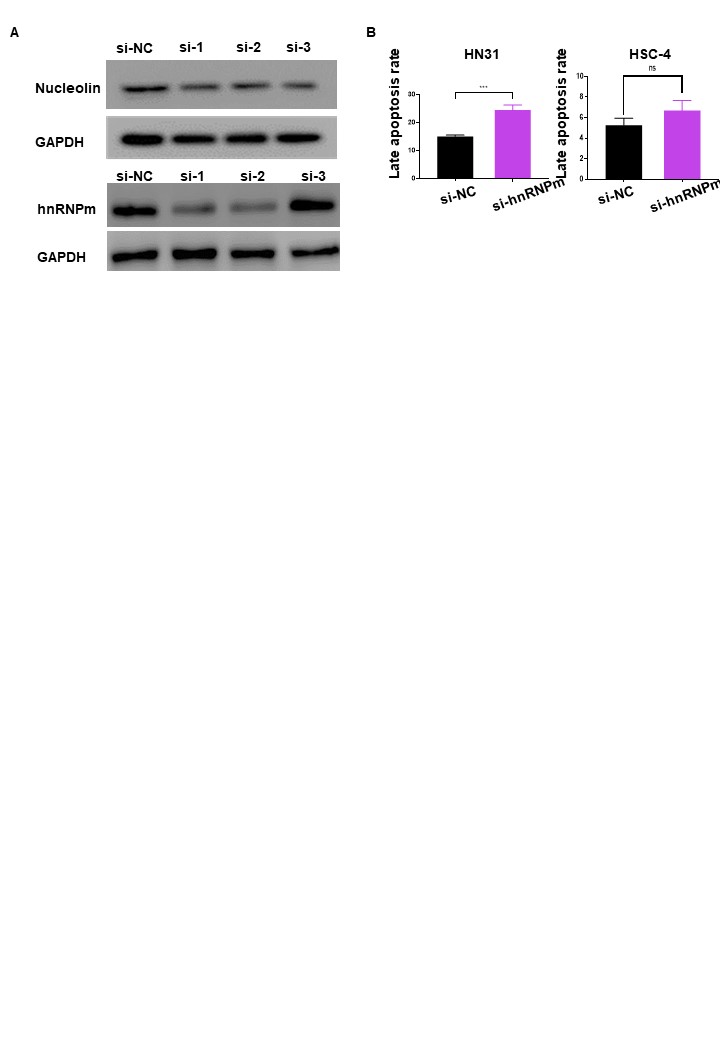

Supplement: Supplementary file 1 [file cancers-15-05351-s001.zip › Figure S3.JPG]

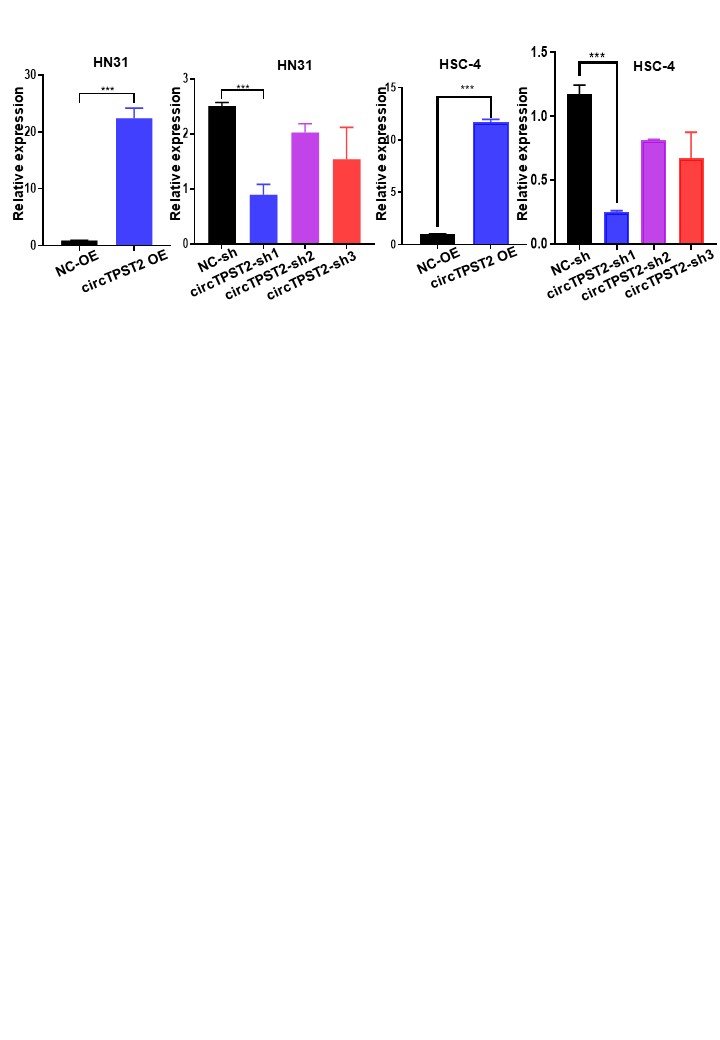

Supplement: Supplementary file 1 [file cancers-15-05351-s001.zip › Figure S4.JPG]

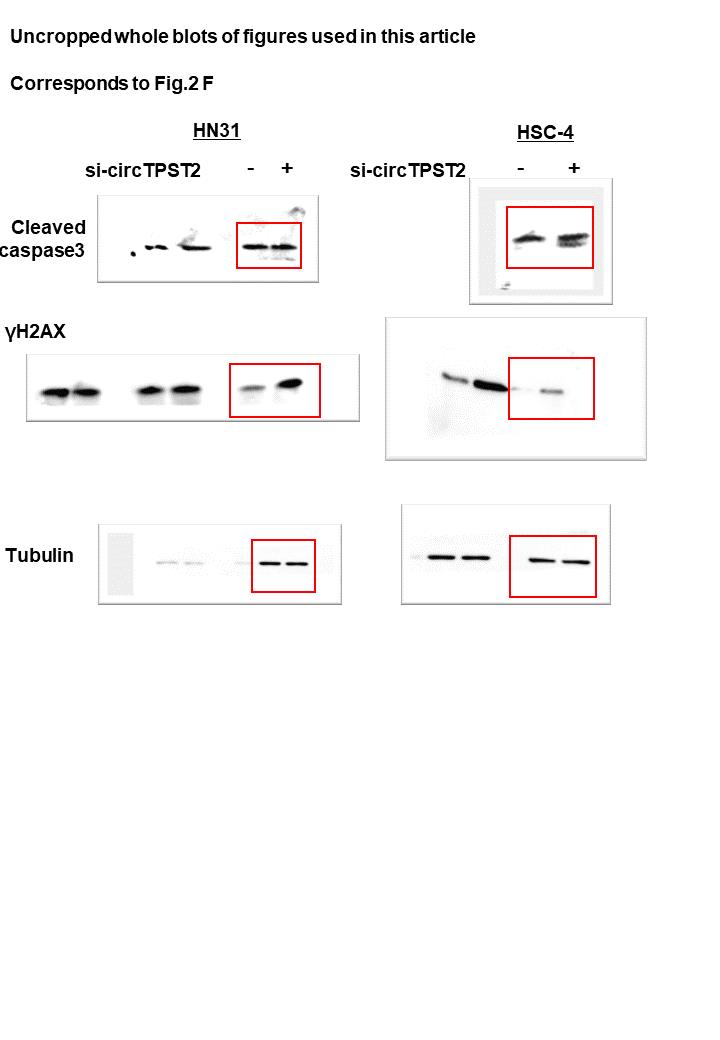

Supplement: Supplementary file 1 [file cancers-15-05351-s001.zip › Figure S5.jpg]

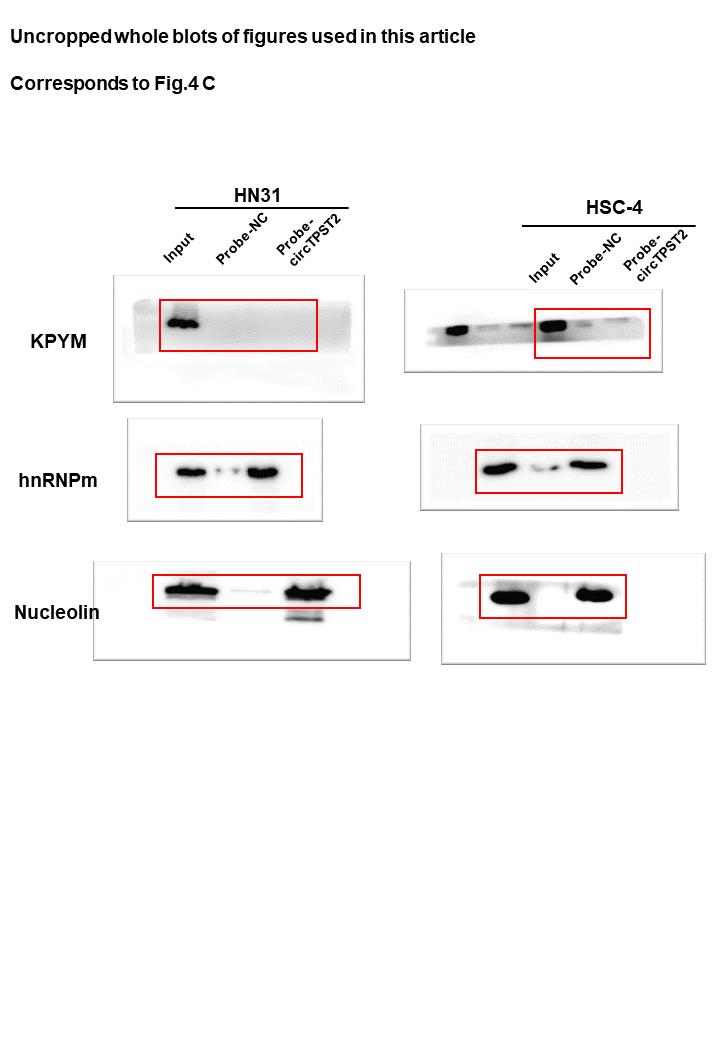

Supplement: Supplementary file 1 [file cancers-15-05351-s001.zip › Figure S6.jpg]

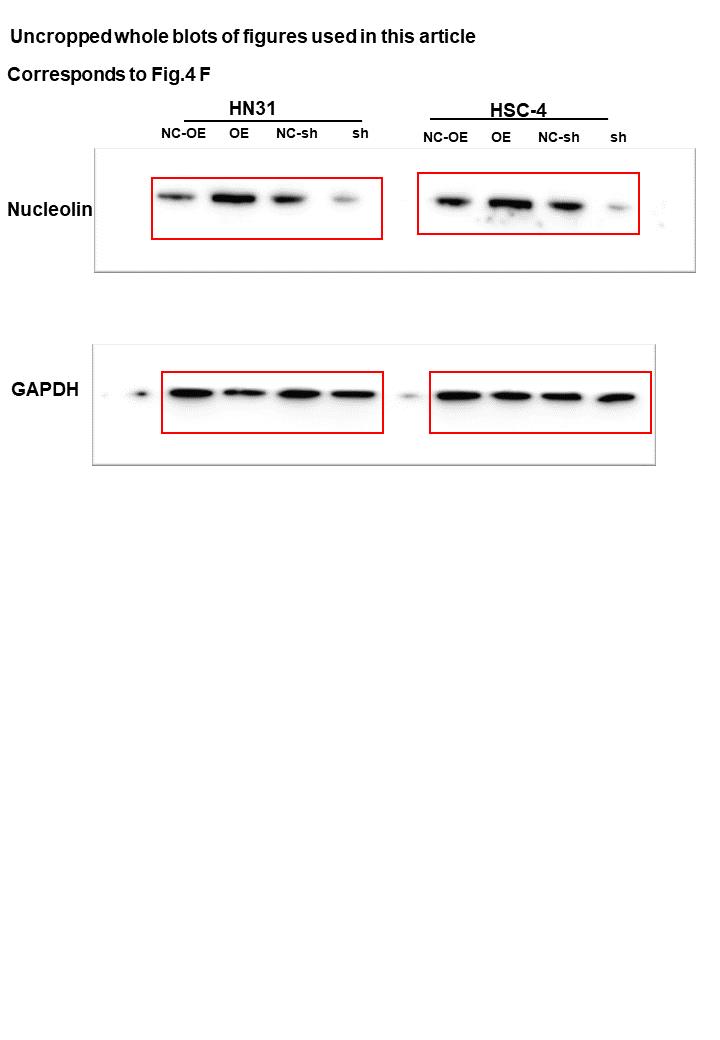

Supplement: Supplementary file 1 [file cancers-15-05351-s001.zip › Figure S7.jpg]

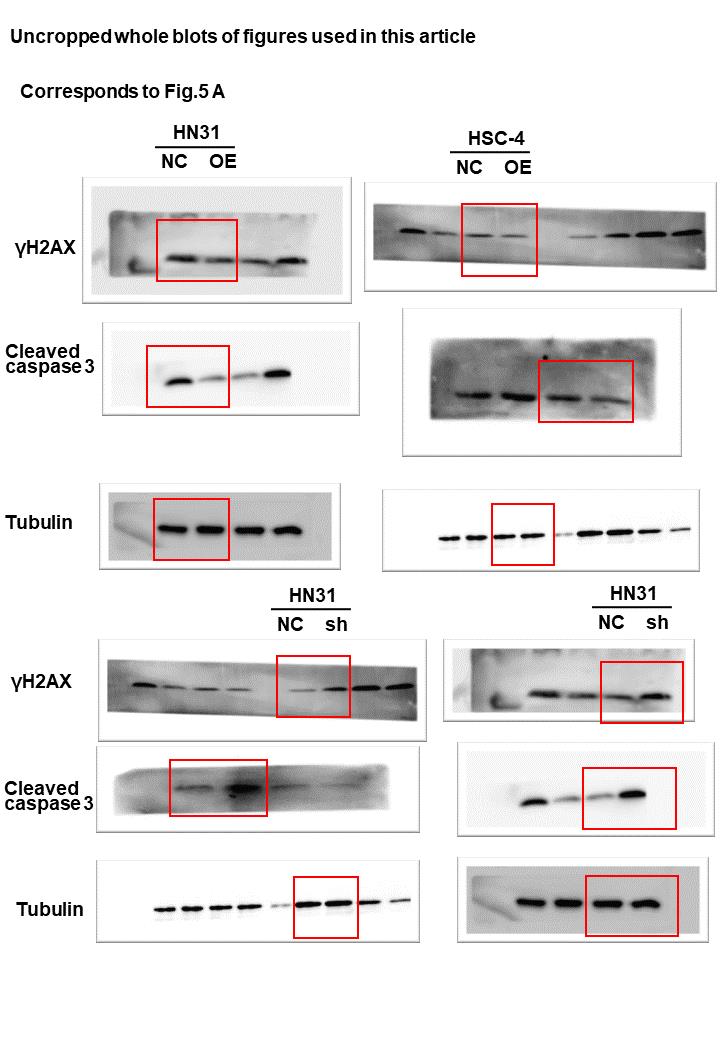

Supplement: Supplementary file 1 [file cancers-15-05351-s001.zip › Figure S8.jpg]

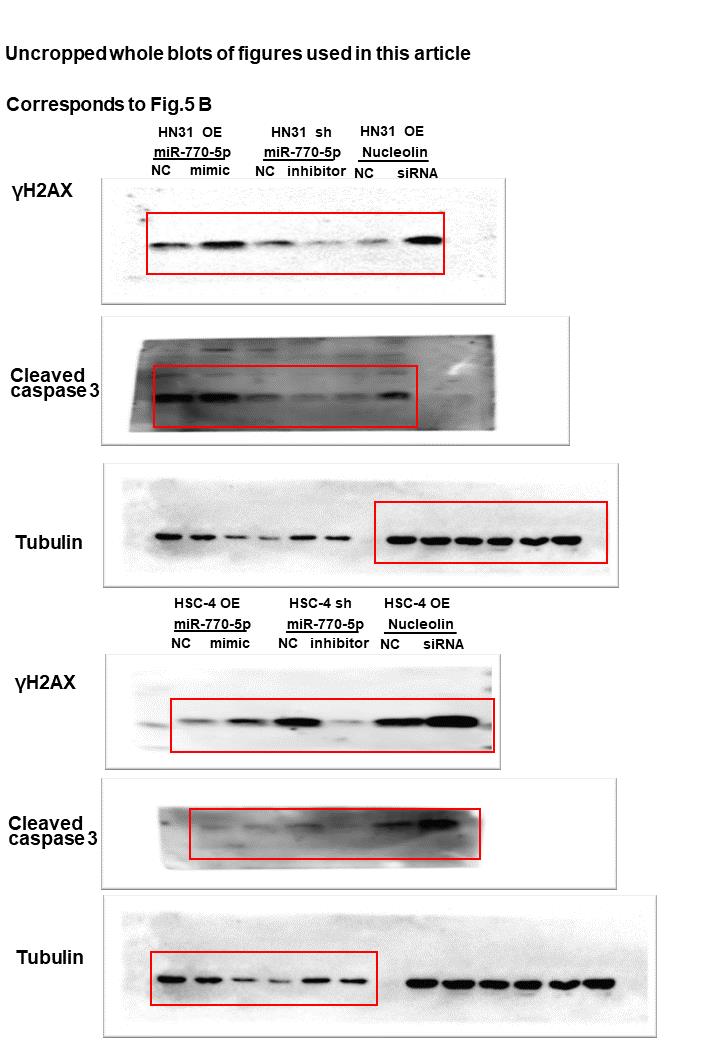

Supplement: Supplementary file 1 [file cancers-15-05351-s001.zip › Figure S9.jpg]
